# Increased Plasma Levels of the Co-stimulatory Proteins CDCP1 and SLAMF1 in Patients With Autoimmune Endocrine Diseases

**DOI:** 10.3389/fimmu.2020.01916

**Published:** 2020-08-24

**Authors:** Louise Magnusson, Daniel Espes, Rosaura Casas, Per-Ola Carlsson

**Affiliations:** ^1^Department of Medical Sciences, Uppsala University, Uppsala, Sweden; ^2^Division of Children and Women Health, Department of Biomedical and Clinical Sciences, Linköping University, Linköping, Sweden; ^3^Department of Medical Cell Biology, Uppsala University, Uppsala, Sweden

**Keywords:** type 1 diabetes, Hashimoto's thyroiditis, Graves' disease, Addison's disease, proximity extension assay, plasma, CDCP1, SLAMF1

## Abstract

Despite that autoimmune diseases share similar immunogenetic mechanisms, studies comparing the protein composition in peripheral blood from patients with autoimmune endocrine diseases are limited. In this study, we applied proximity extension assay to measure proteins related to signaling and interactions within the immune system in peripheral blood from patients with new-onset (N-T1D) and long-standing (L-T1D) type 1 diabetes, Hashimoto's thyroiditis (HT), Graves' disease (GD), and autoimmune Addison's disease in addition to healthy controls (HC). Proteins in plasma and supernatants from cultured PBMC were measured by using a 92-plex Olink® INFLAMMATION panel. Soluble CDCP1 was more abundant in plasma from patients with N-T1D, L-T1D, HT, and GD than in HC. The L-T1D and HT groups had elevated plasma levels of SLAMF1 compared with HC. Patients and HC could not be distinguished by their protein composition in PBMC supernatants. The high-throughput multiplex technology enabled us to detect two low-abundant proteins that have been gradually connected to autoimmune diseases. Our study provides novel associations between CDCP1, SLAMF1, and autoimmune endocrine diseases, which might reflect a higher degree of inflammation and lymphocyte activation.

## Introduction

The concepts “mosaic of autoimmunity” and “autoimmune tautology” declare that several features underlying autoimmunity are shared between diseases (1, 2). The proposed model pertains all autoimmune conditions, although genetic, hormonal, immunological, and environmental factors can interact differently and thus determine the final disease. A large proportion of patients with type 1 diabetes (T1D), Hashimoto's thyroiditis (HT), Graves' disease (GD), and autoimmune Addison's disease (AD) develop poly-autoimmunity and/or Autoimmune Polyendocrine Syndrome (3–7), inferring that such patients have similar immunological deviations. In T1D, loss of β-cell mass or function leads to reduced insulin production and hyperglycemia. HT and GD are autoimmune diseases of the thyroid gland that cause hypo- and hyperthyroidism, respectively. The loss of aldosterone and cortisol production in AD is caused by immune-mediated destruction within the adrenal glands. By measuring cytokines and chemokines in various sample matrices, it has been hypothesized that T1D, HT, GD, and AD are mainly mediated by pro-inflammatory and Th1-biased immune responses (8–14). However, concurrent Th2-/Th17-biased immune responses have also been reported (12, 15, 16). An issue with abovementioned studies is that no or few simultaneous comparisons between diseases have been made, as patients with only one autoimmune condition have been mostly recruited. In addition, a limited number of analytes have been assessed despite that immune responses comprise a complex network of protein interactions.

A novel approach to simultaneously measure more than 90 analytes in different sample matrices is proximity extension assay (PEA). This technology combines a dual-recognition immunoassay and quantitative PCR, yielding improved assay sensitivity and multiplexing capacity (17, 18). PEA has been employed to investigate immunological signatures related to cancer (19), pregnancy and infancy (20, 21), and age (22). Although two studies addressing GABAergic signaling in T1D (23) and cardiovascular risk in AD (24) have used this technology, immune-related proteins in peripheral blood from patients with autoimmune endocrine diseases have not been previously compared. Thus, we applied high-throughput multiplex PEA to interrogate the protein composition in plasma and PBMC supernatants from patients with T1D, HT, GD, and AD. Although the patients could not be distinguished by the overall protein composition in either sample matrix, plasma levels of two analytes related to inflammation and lymphocyte activation were elevated in patients with T1D, HT, and GD.

## Materials and Methods

### Study Design and Participants

Patients with new-onset (N-T1D) and long-standing T1D (L-T1D), HT, GD, and AD in addition to healthy controls (HC) were recruited at Uppsala University Hospital between January 2017 and June 2018. All participants were 18–50 years old and had no signs of infections, malignancies, or other diseases at inclusion. Patients had only one known autoimmune endocrine disease and did not present derangements in hormone production and autoantibodies for any of the other studied diseases. HC had no first-degree relatives with autoimmune endocrine diseases, no derangements in hormonal function and were negative for the tested disease-specific autoantibodies. All patients were medicated with disease-specific substitution treatments: intensive insulin therapy for T1D, levothyroxine for HT, block-replace therapy (thiamazole with addition of levothyroxine after 4 weeks) for GD and hydrocortisone for AD. Patients with GD had not been previously treated with radioiodine or ablative surgery.

Creatinine (μmol/L), fasting glucose (mmol/L), glycated hemoglobin (HbA1c, mmol/mol), C-peptide (nmol/L), thyroid-stimulating hormone (mIE/L), free thyroxine (pmol/L), free triiodothyronine (pmol/L), serum cortisol (nmol/L), anti-glutamic acid decarboxylase (IE/mL), anti-tyrosine phosphatase like protein islet antigen-2 (kE/L), anti-thyroid peroxidase (kIE/L), thyroid-stimulating immunoglobulins (E/L), and anti-21-hydroxylase (kE/L) were measured at the Clinical Chemistry and Pharmacology Laboratory, Uppsala University Hospital, Sweden. Table 1 summarizes the characteristics of patients and HC. The sex distribution was similar between the groups, whereas patients with N-T1D were younger than individuals in the L-T1D and AD groups (*p* < 0.05). This study was approved by the Regional Research Ethical Committee in Uppsala (Dnr 2014/485) and was consistent with The Declaration of Helsinki. All participants gave their written informed consent prior to inclusion in the study.

**Table 1:** Characteristics of patients and healthy individuals.

**Parameter**	**HC** **(*n* = 16)**	**N-T1D** **(*n* = 7)**	**L-T1D** **(*n* = 9)**	**HT** **(*n* = 8)**	**GD** **(*n* = 8)**	**AD** **(*n* = 8)**
Age (y)	28.8 ± 1.4	23.3 ± 1.0	34 ± 2.9	32 ± 2.9	33.8 ± 3.2	36.3 ± 2.5
Sex (% male)	50	71	56	25	38	75
Disease duration (y)	NA	0.3 ± 0.03	19.1 ± 2.4	4.2 ± 1.0	0.5 ± 0.1	6.3 ± 1.8
BMI (18.5–25 kg/m^2^)	23.1 ± 0.7	21.1 ± 0.6	26.2 ± 1.4	24.3 ± 1.1	26.2 ± 1.7	25.4 ± 1.6
Creatinine (60–105 μmol/L)	76.9 ± 3.8	65.0 ± 5.2	70.5 ± 3.2	67.0 ± 2.0	66.1 ± 4.2	85.5 ± 4.1
F-Glucose (4.0–6.0 mmol/L)	5.6 ± 0.1	11.3 ± 2.0	11.2 ± 1.0	5.9 ± 0.2	5.3 ± 0.3	5.2 ± 0.2
HbA1c (27–42 mmol/mol)	31.7 ± 0.6	75.4 ± 11.0	59.2 ± 3.0	32.5 ± 1.0	31.3 ± 1.3	32.9 ± 0.9
TSH (0.4–4.0 mIE/L)	2.0 ± 0.2	3.8 ± 1.0	2.3 ± 0.2	5.9 ± 2.4	0.9 ± 0.7	3.0 ± 0.4
T3 (3.1–6.8 pmol/L)	5.1 ± 0.1	5.4 ± 0.1	4.8 ± 0.2	4.5 ± 0.2	6.1 ± 1.0	6.3 ± 0.3
T4 (12.0–22.0 pmol/L)	15.6 ± 0.4	15.2 ± 0.8	15.0 ± 0.4	16.0 ± 0.9	21.1 ± 2.3	16.3 ± 1.0
S-Cortisol (220–650 nmol/L)	532 ± 67	497 ± 136	466 ± 25	425 ± 27	336 ± 24	526 ± 64

*Age, sex distribution, disease duration, BMI, and clinical parameters at the time of sampling were assessed in patients with new-onset (N-T1D) and long-standing (L-T1D) type 1 diabetes, Hashimoto's thyroiditis (HT), Graves' disease (GD), and autoimmune Addison's disease (AD) as well as healthy controls (HC). Normal ranges for clinical parameters are indicated. All values are given as mean ± SEM. HbA1c, glycated hemoglobin; NA, not applicable; TSH, thyroid-stimulating hormone; T3, free triiodothyronine; T4, free thyroxine; y, years*.

### Blood Sampling and Preparation of PBMC

Peripheral venous blood was collected in three Vacuette® sodium-heparin tubes à 9 mL (Greiner Bio-One; Austria) in the morning (8–10 A.M). All participants had been fasting since 10 P.M the night before sampling. Blood samples were sent to the Pediatric research laboratory, Linköping University, at room-temperature and were processed within 27 h after sample collection. Plasma was separated from heparinized blood and stored at −70°C prior to analysis. PBMC were isolated by performing density-gradient centrifugation with Leucosep® tubes (Greiner Bio One; Austria) and Ficoll-Paque™ PLUS (density 1.077 ± 0.001 g/mL, GE Healthcare; UK) according to manufacturer's instructions. Cells were washed twice in RPMI 1640 w/o L-glutamine (Gibco; US) supplemented with 2 % (v/v) heat-inactivated fetal calf serum (Gibco; US).

### Secretion Assay

Cells were washed and diluted to a concentration of 1 × 10^6^ cells/mL in supplemented stimulation medium [serum-free AIM-V® medium (Gibco; US) with 0.02% (v/v) β-mercaptoethanol (Sigma-Aldrich; US)]. PBMC (5 × 10^5^/condition) were left unstimulated and cultured with Dynabeads® Human T-Activator CD3/CD28 (Gibco; US) at a bead/cell-ratio of 1:20 in polypropylene tubes (12 × 75 mm, Sarstedt; Germany). After seven days of culture at 37°C and 5% CO_2_, supernatants were retrieved and stored at −70°C prior to analysis. Three participants were excluded from this experiment due to an insufficient number of PBMC during the isolation procedure.

### Proximity Extension Assay and Quality Control

Proteins in plasma and cell supernatants were measured by using high-throughput multiplex PEA. Undiluted samples were sent on dry ice to the Analysis Service at Olink Proteomics AB in Uppsala, Sweden, for analysis. Four supernatant samples from stimulated PBMC were diluted 2-, 10-, and 20-fold to assess possible Hook-effects. A validated Olink® INFLAMMATION panel was used to simultaneously measure 92 analytes associated with immune responses. Development and optimization of PEA have been described elsewhere (17, 18). Briefly, thawed samples were incubated with 92 pairs of oligonucleotide-labeled detection antibodies. Upon specific binding of paired detection antibodies to a target (dual protein-recognition step), juxta-positioned complementary DNA oligonucleotides could hybridize. Hybridized oligonucleotides then served as templates for DNA polymerase to synthesize dsDNA barcodes (extension step). In the detection step, dsDNA barcodes were amplified by PCR and then quantified by high-throughput microfluidic qPCR (BioMark™ HD System, Fluidigm; US) in a 96.96 Dynamic Array Integrated Fluidic Circuit (Fluidigm; US).

Four internal controls were spiked at a pre-determined concentration into all samples to evaluate the technical assay performance and sample quality. Two non-human proteins (incubation controls) monitored all assay steps, whereas an antibody conjugated to two complementary oligonucleotides (extension control) monitored the extension, amplification, and qPCR steps. Lastly, one synthetic dsDNA amplicon (detection control) monitored the last two PCR steps. Standard deviations for both incubation controls and the detection control were calculated to evaluate assay runs. For the assessment of sample quality, median values and standard deviations for one incubation control and the detection control were calculated in each sample. To determine the lower limit of detection for each analyte, matrix-specific negative controls were used in triplicates. An inter-plate control, comprising a pool of 92 extension controls, was added in triplicates for data normalization. External sample controls were added in duplicates for calculation of intra- and inter-assay coefficient of variance (Supplementary Table 1).

### Data Acquisition, Analysis, and Statistics

Quantitation cycle (Cq)-values for each analyte were acquired and further processed in Fluidigm Real-Time PCR analysis software. In order to reduce technical and inter-plate variations, Cq-values for each analyte were normalized with Cq-values from the extension and inter-plate controls. Normalized Cq-values were then transformed into a relative arbitrary unit on a log2 scale, called Normalized Protein eXpression (NPX), by using pre-determined correction factors for each analyte. Although NPX directly correlates with initial protein concentrations, no comparisons of absolute protein levels can be made. Data for unstimulated and stimulated supernatants were analyzed separately. The induced secretion was also assessed by calculating the pairwise difference of NPX between stimulated and unstimulated samples: NPX_stim_-NPX_unstim_. Principal Component Analysis was applied to visualize similarities and differences in multivariate datasets between plasma and supernatant samples, respectively. One-way ANOVA adjusted for multiple testing with the Benjamini-Hochberg method, followed by Tukey's *post-hoc* test for significant assays, were applied for analysis of immunological data (α = 0.05). One-way ANOVA with Tukey's test for multiple comparisons and chi-square test were used to analyze clinical data (α = 0.05). Two-tailed non-parametric Spearman correlation was applied to investigate covariations between immunological and clinical data (α = 0.05), along with linear regression to find the best-fit line.

## Results

Plasma and supernatants from cultured PBMC were collected in order to compare the peripheral protein composition between patients with T1D, HT, GD, and AD as well as HC. We employed PEA to measure 92 analytes related to signaling and interactions within the immune system, such as cytokines, chemokines, enzymes, and shed surface receptors. All plasma samples passed the quality control, whereas four supernatant samples were excluded from the data analysis (Supplementary Figures 1A,B). Analytes that were detected in at least 25 % of the samples were analyzed in this study: 81 proteins in plasma and 67 proteins in supernatants (Supplementary Table 2).

### Soluble Isoforms of CDCP1 and SLAMF1 Were More Abundant in Plasma From Patients With T1D, HT, and GD

**Figure 1:**
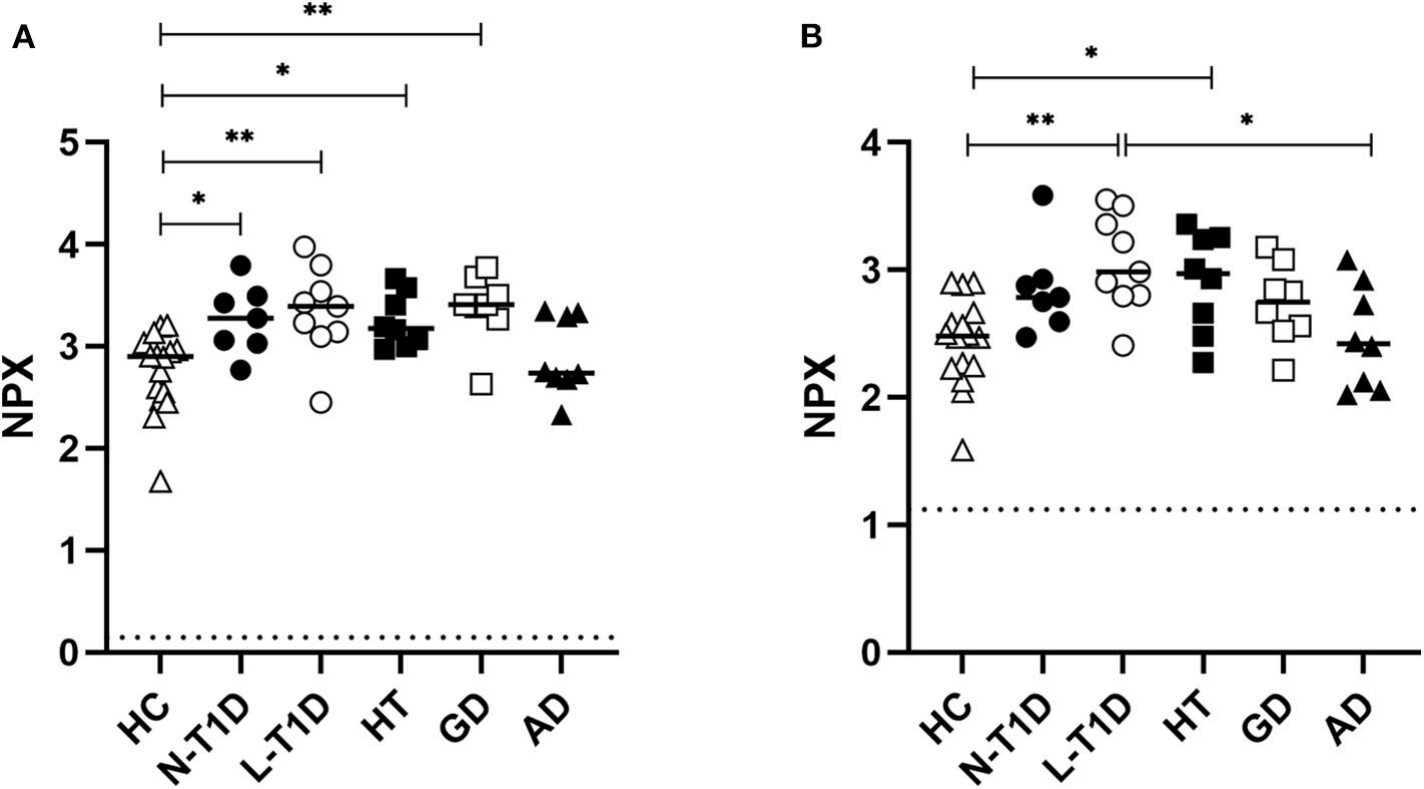
Plasma levels of soluble CDCP1 and SLAMF1 were elevated in patients with autoimmune diabetes, hypo-, and hyperthyroidism. Plasma from patients with new-onset (N-T1D, *n* = 7) and long-standing (L-T1D, *n* = 9) type 1 diabetes, Hashimoto's thyroiditis (HT, *n* = 8), Graves' disease (GD, *n* = 8), and autoimmune Addison's disease (AD, *n* = 8) as well as healthy controls (HC, *n* = 16) was analyzed by proximity extension assay. The abundance of **(A)** CDCP1 and **(B)** SLAMF1 is shown as Normalized Protein eXpression (NPX), a relative arbitrary unit on a log2 scale. The individual and median NPX-values are indicated. Dotted lines represent the lower limit of detection for CDCP1 (0.147 NPX) and SLAMF1 (1.12 NPX). One-way ANOVA adjusted for multiple testing with the Benjamini-Hochberg method, followed by Tukey's *post-hoc* test, were applied for statistical analysis, *p < 0.05, **p < 0.01.

**Figure 2:**
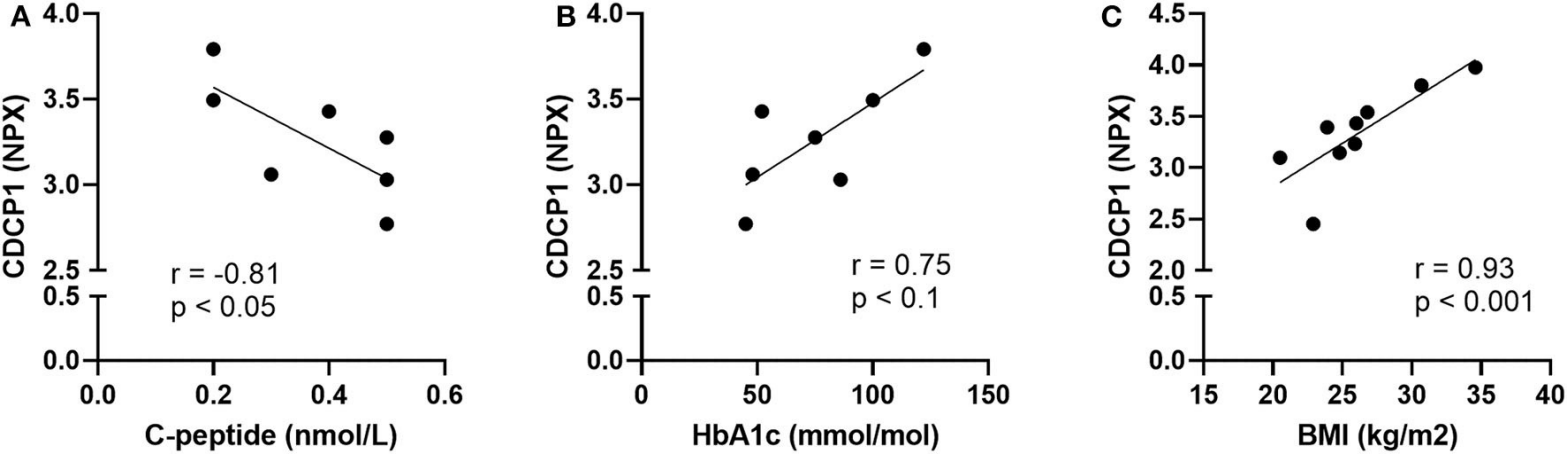
Plasma levels of CDCP1 correlated with clinical parameters for diabetic patients. Plasma from patients with new-onset (N-T1D) and long-standing (L-T1D) type 1 diabetes, Hashimoto's thyroiditis, Graves' disease, and autoimmune Addison's disease as well as healthy controls was analyzed by proximity extension assay. The abundance of CDCP1, shown as Normalized Protein eXpression (NPX), correlated with **(A)** fasting C-peptide concentrations and **(B)** glycated hemoglobin (HbA1c) in patients with N-T1D (*n* = 7). The plasma levels of CDCP1 correlated with **(C)** body mass index in patients with L-T1D (*n* = 9). Dots represent independent patient samples and best-fit lines were plotted by applying linear regression. Correlation coefficients and *p*-values are presented for each graph, which were obtained by applying two-tailed non-parametric Spearman correlation.

Principal Component Analysis was applied to identify possible patterns in the plasma protein composition of patients with autoimmune endocrine diseases. Patients and healthy individuals could not be distinguished by their protein composition in plasma, as there were no differences between samples (Supplementary Figure 2). However, statistical analysis of each analyte revealed that CDCP1 was more abundant in the N-T1D (*p* = 0.04), L-T1D (*p* = 0.01), HT (*p* = 0.03), and GD (*p* = 0.004) groups than in HC (Figure 1A). Patients with L-T1D also had elevated plasma levels of SLAMF1 compared with HC (*p* = 0.002) and the AD group (*p* = 0.02), whereas SLAMF1 was more abundant in plasma from patients with HT than from HC (*p* = 0.049, Figure 1B). When analyzing the covariation between these plasma proteins and clinical variables, levels of CDCP1 in patients with N-T1D were inversely correlated with fasting C-peptide concentrations (Figure 2A, *p* = 0.048). In contrast, increased levels of CDCP1 in the N-T1D group appeared to be associated with increased HbA1c (Figure 2B, *p* = 0.07). For the L-T1D group, a positive covariation between CDCP1 levels and body mass index was found (Figure 2C, *p* = 0.0007). Despite that 26 other analytes seemed to differ between the groups, these alterations were non-significant after adjustment for multiple testing (Table 2).

**Table 2:** Immune-related proteins in plasma.

**Abbreviation**	***p*-value**	**Adjusted *p*-value**
*CDCP1*	*0.00*	*0.03*
*SLAMF1*	*0.00*	*0.04*
Beta-NGF	0.00	0.10
DNER	0.01	0.17
CD5	0.01	0.17
CCL11	0.02	0.17
IL-12B	0.02	0.17
IL-18R1	0.02	0.17
CST5	0.02	0.17
CXCL6	0.02	0.19
MMP-10	0.03	0.19
IL-20RA	0.03	0.19
FGF-23	0.03	0.20
CCL25	0.04	0.23
CCL19	0.05	0.23
TNFRSF9	0.05	0.23
GDNF	0.05	0.23
OPG	0.05	0.25
TRANCE	0.06	0.25
SCF	0.06	0.25
CXCL11	0.06	0.25
IL-17A	0.07	0.25
CXCL10	0.08	0.25
CXCL1	0.08	0.25
IL-24	0.08	0.25
CCL20	0.08	0.25
LIF-R	0.08	0.25
CX3CL1	0.09	0.25

*Twenty-eight analytes in plasma appeared to differ between patients with new-onset (n = 7) and long-standing (n = 9) type 1 diabetes, Hashimoto's thyroiditis (n = 8), Graves' disease (n = 8), and autoimmune Addison's disease (n = 8) as well as healthy controls (n = 16). Only two proteins were significantly different after adjustment for multiple testing with the Benjamini-Hochberg method (italics)*.

### The Secretome of PBMC Was Similar Between Patients and Healthy Individuals

A clear separation between supernatants from unstimulated and stimulated PBMC was observed (Supplementary Figure 3), illustrating that the secretome of PBMC had changed after incubation with anti-CD3/CD28 for seven days. There was no distinction between patients and HC based on the protein composition in supernatants, irrespective of culture condition (Supplementary Figures 4A,B). Furthermore, there were no differences in relative protein levels or the differential protein secretion between the groups (Table 3).

**Table 3:** Immune-related proteins in cell supernatants.

**Abbreviation**	***p*-value**	**Adjusted *p*-value**
**Unstimulated**		
CCL28	0.02	0.41
MCP-3	0.02	0.41
CCL23	0.02	0.41
CXCL1	0.08	0.97
**Stimulated**		
TWEAK	0.04	0.82
CCL28	0.05	0.82
**NPX**_**stim**_**-NPX**_**unstim**_		
PD-L1	0.00	0.10
LAP TGF-beta-1	0.08	0.87

*PBMC were isolated from patients with new-onset (N-T1D) and long-standing (L-T1D) type 1 diabetes, Hashimoto's thyroiditis (HT), Graves' disease (GD), and autoimmune Addison's disease (AD) as well as healthy controls (HC). PBMC were left unstimulated (N-T1D, n = 6; L-T1D, n = 9; HT, n = 7; GD, n = 8; AD, n = 7; HC, n = 15) and stimulated with anti-CD3/CD28 Dynabeads (N-T1D, n = 6; L-T1D, n = 9; HT, n = 6; GD, n = 7; AD, n = 7; HC, n = 15). The induced protein secretion (NPX_stim_-NPX_unstim_) was thereafter calculated. No proteins were significantly different after adjustment for multiple testing with the Benjamini-Hochberg method*.

## Discussion

By applying high-dimensional mass cytometry, we previously identified alterations of phenotypically distinct cell subsets in patients with N-T1D, HT, and AD (25). In this study, we continued our interrogation on shared and diverging peripheral immunological features in patients with T1D, HT, GD, and AD. For this purpose, we used a high-throughput multiplex immunoassay to analyze proteins related to signaling and interactions within the immune system in plasma and cell supernatants. The protein profiles in both sample matrices were in general similar between patients with autoimmune endocrine diseases and HC. However, plasma levels of two low-abundant proteins were elevated in several patient groups.

We detected elevated plasma levels of CDCP1 (CD318) in patients with N-T1D, L-T1D, HT, and GD. CDCP1 is mainly expressed on the surface of mesenchymal tissue cells and non-professional antigen-presenting cells, but a soluble isoform has been identified (26, 27). This protein has been extensively associated with cell adhesion and tumor biology (28, 29), but an increasing body of evidence suggests that it modulates T cell responses upon activation. Studies have shown that CDCP1 interacts with the membrane-distal domain of CD6, which is expressed on T cells and acts as a co-stimulatory receptor within the immunological synapse (26, 27, 30). It is therefore interesting that aberrant CDCP1-singaling has been previously associated with autoimmunity and inflammation. IFN-γ can induce the expression of this ligand on mesenchymal tissue cells (26, 27), indicating that an existent pro-inflammatory milieu can facilitate further lymphocyte activation. Enyindah-Asonye et al. have also provided mechanistic insights on the involvement of CDCP1 in autoimmune diseases (27). In an animal model for multiple sclerosis, knock-out of *Cdcp1* led to improved clinical scores and reduced infiltration of CD4^+^ T cells producing IFN-γ and IL-17. Moreover, membrane-bound and soluble isoforms of this protein were more abundant within the synovium from patients with rheumatoid arthritis compared with control groups. As there was a gradient of soluble CDCP1 between sera and synovial fluid, Enyindah-Asonye et al. showed that this protein was chemotactic for human peripheral T cells. Whereas this is the first study, to our knowledge, that reveals an association between CDCP1, HT, and GD, a previous study also found elevated CDCP1 levels in plasma from patients with L-T1D (23). In this study, we report that CDCP1 in plasma correlated with parameters for insulin production as well as glycemic and metabolic control. The inverse correlation between soluble CDCP1 and fasting C-peptide in patients with N-T1D suggests that a reduced insulin production is connected to inflammation, possibly within the pancreas, at disease onset. Naturally, a decreased insulin release from the pancreas entails a challenge for patients to maintain glycemic control, as indicated by the positive correlation between CDCP1 and HbA1c. As CDCP1 was only associated with body mass index in the L-T1D group, it seems that the ability to maintain an optimized metabolic control has a substantial effect on whole-body inflammation at later stages of T1D (31, 32). We hypothesize that increased plasma levels of soluble CDCP1 reflect an ongoing inflammatory response in patients with T1D, HT, and GD due to an increased shedding from tissue cells. Hypothetically, a gradient of the soluble isoform might stimulate T cells to migrate into affected endocrine glands and promote T cell-differentiation.

SLAMF1 (CD150) was also more abundant in plasma from patients with L-T1D and HT than HC. This 70 kDa-protein is a member of the immunoglobulin superfamily and exists in membrane-bound and soluble isoforms (33, 34). SLAMF1 is a co-stimulatory receptor that co-localizes with CD3 upon T cell activation and affects downstream signaling (35). Both isoforms are up-regulated upon activation and then constitutively expressed on memory T and B cells (33, 34). SLAMF1-ligation can induce the secretion of IFN-γ, but not IL-4 and IL-5, from uncommitted Th-, Th1-, and Th2 clones (33). Another *in vitro* study reported that ligation of this co-stimulatory receptor increased immunoglobulin production from peripheral B cells (34). This protein has been previously associated with autoimmunity, as an increased expression of SLAMF1 on peripheral and tissue-resident lymphocytes from patients with rheumatoid arthritis and systemic lupus erythematous have been reported (36, 37). The expression of SLAMF1 on peripheral lymphocytes has also been studied in patients with autoimmune thyroid diseases, where Vitales-Noyola et al. demonstrated a higher frequency of SLAMF1^+^CD4^+^ T cells in patients than in HC (38). A higher abundance of SLAMF1 in plasma from the HT group might be associated with our previous observation that patients with HT had elevated frequencies of plasmablasts and CD11c^+^CD27^−^T-bet^+^ B cells (25). As these subsets comprise antibody-producing memory B cells (39, 40), a higher abundance of soluble SLAMF1 might indicate that plasmablasts and CD11c^+^CD27^−^T-bet^+^ B cells from patients with HT are activated and continuously stimulated to produce autoantibodies.

In this study, we were not able to detect any diverging peripheral protein profile in the AD group. This disease has been less scrutinized, as it is a rare condition and patients are often affected by co-morbidities (41). AD has been associated with Th1-biased immune responses, as indicated by elevated serum or plasma levels of CXCL9, CXCL10, and CXCL11 (8, 9, 42, 43). However, these alterations do not seem to be applicable to all patients and studies have failed to show differences at a cellular level (8, 9, 43). In our study, the lack of alterations in the AD group can be explained by two mechanisms. Firstly, patients were medicated with adjusted doses of hydrocortisone according to the conventional substitution treatment. It has been shown that glucocorticoids selectively inhibit IFN-γ and IRF-1 expression in T cells (44) and suppress IFN-γ-induced chemokine secretion from human adrenocortical cells (9). It is recognized that glucocorticoids promote Th2-biased immune responses (44–46), suggesting that the treatment might have modulated preceding Th1-biased immune responses in patients included in our study. Secondly, clinical symptoms of AD become manifested when >90% of functional tissue has been destroyed (47), implying that a lack of autoantigens would lead to reduced inflammation and activation of CD45^+^ cells.

PEA was applied in our study as several immune-related analytes could be measured simultaneously in a single sample. Thus, this discovery-oriented technology allowed us to assess well-known and exploratory proteins for different autoimmune endocrine diseases in parallel. In addition, PEA precluded inter-manufacturer variations that could have occurred if ELISA or other multiplex assays would have been used to measure several analytes. It is surprising that we did not observe pro-inflammatory signatures commonly associated with autoimmune endocrine diseases in either plasma or cell supernatants. As some analytes were excluded from the data analysis due to low detectability, it is important to consider whether a targeted assay would have been sufficient. For example, a clinical trial addressing celiac disease tested PEA on a small number of samples, but changed into a bead immunoassay and electrochemiluminescence to circumvent detectability issues for proteins of interest (48). Secondly, statistical analysis of 92 analytes between several groups entails a considerable issue with multiple testing and comparisons. Although adjustments reduce type I errors, they might not always be applicable as possible true positive findings are neglected (49). As this study was mainly exploratory, our aim was not to reject a universal null hypothesis but to assess each analyte in its own right. We acknowledge that the sample size is a limitation of the study. The eligibility criteria were restricted to patients with only one autoimmune disease, who were well-controlled at the time of sampling and without other co-morbidities. Patients with autoimmune endocrine diseases have unfortunately a propensity to develop poly-autoimmunity and other pathological conditions (3, 5, 6), which makes it difficult recruit a large number of well-defined patients without extending the sampling period. The last consideration is that PEA is a homogenous assay (17), meaning that possible interfering components in plasma and supernatants remained during the entire assay. PEA has however a high sensitivity limit due to signal amplification by PCR, which should reduce sample matrix interferences.

In conclusion, PEA enabled us to detect two low-abundant proteins that have been gradually connected to autoimmune diseases. An increased abundance of CDCP1 and SLAMF1 in plasma from patients with T1D, HT, and GD might reflect a higher degree of inflammation and lymphocyte activation. Our study provides novel associations between CDCP1, SLAMF1, and autoimmune endocrine diseases, but functional studies on PBMC are required to elucidate the mechanistic roles of these proteins.

## Data Availability Statement

The datasets generated for this study are available on request to the corresponding author.

## Ethics Statement

The studies involving human participants were reviewed and approved by the Regional Research Ethical Committee in Uppsala. The patients/participants provided their written informed consent to participate in this study.

## Author Contributions

DE and P-OC were responsible for study conception, recruitment and handling of clinical data. LM and RC designed the immunological experiments. LM performed the cell isolation, secretion assay, sample preparation, data analysis, and data interpretation. LM wrote the manuscript, which was revised by DE, RC, and P-OC. Submission of manuscript was approved by all authors.

## Conflict of Interest

The authors declare that the research was conducted in the absence of any commercial or financial relationships that could be construed as a potential conflict of interest.
