# Supplementary material for: Increased Plasma Levels of the Co-stimulatory Proteins CDCP1 and SLAMF1 in Patients With Autoimmune Endocrine Diseases
Source: Front Immunol. 2020 Aug 24;11:1916. doi: 10.3389/fimmu.2020.01916 (PMC7476208; doi:10.3389/fimmu.2020.01916)
Supplement: Supplementary file 1 [file Presentation_1.PPTX]

## Slide 1
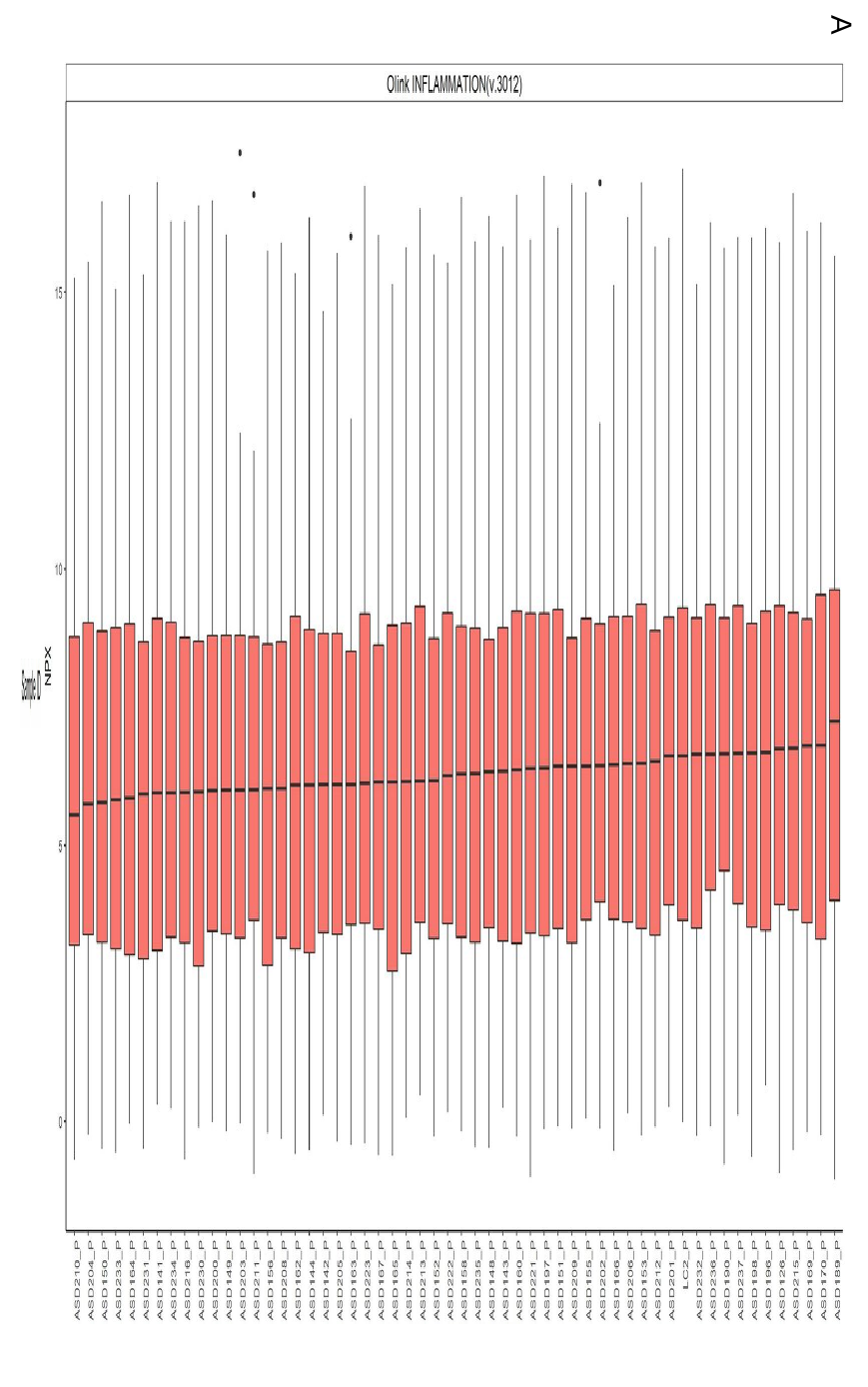

A

## Slide 2
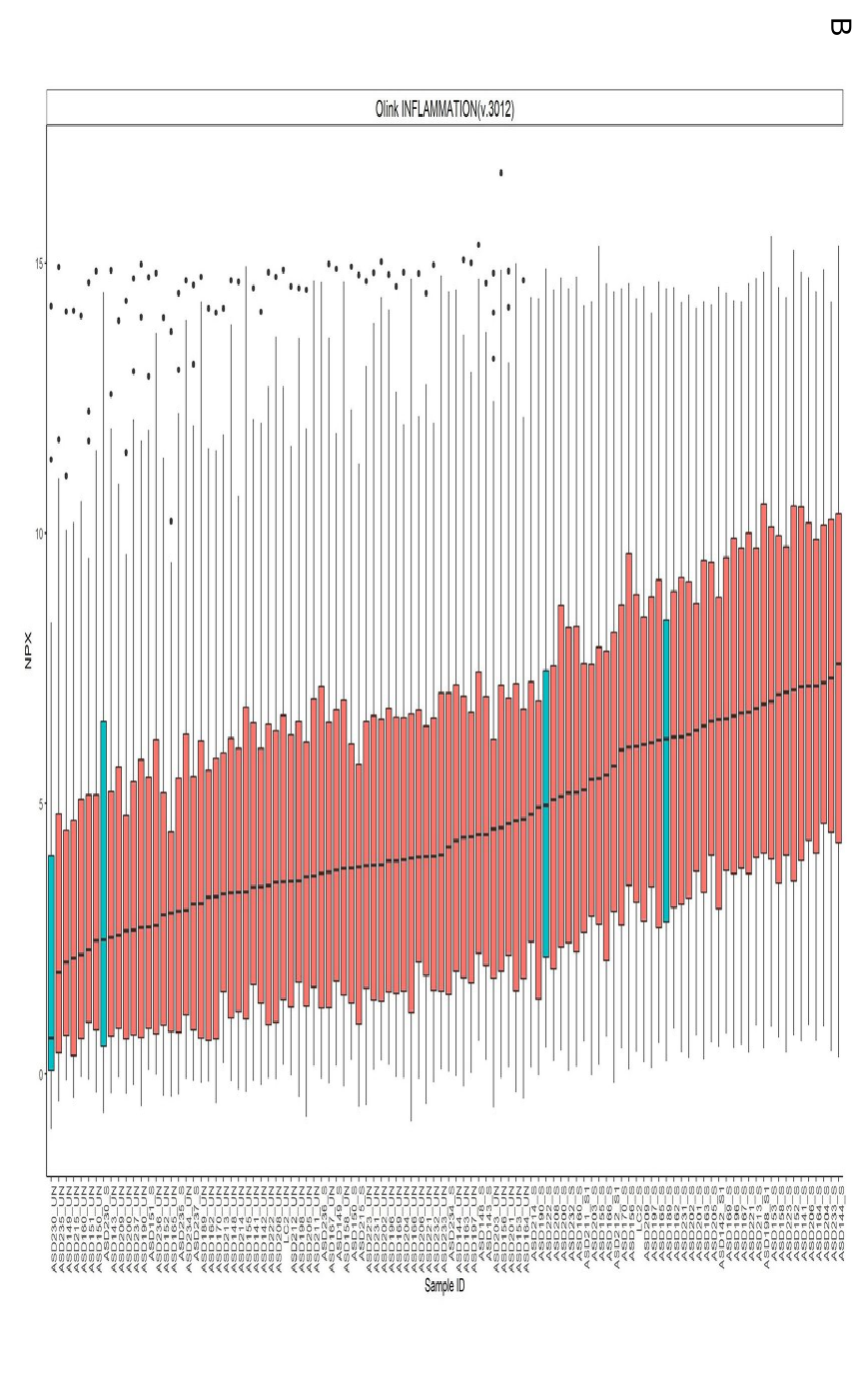

B

## Slide 3
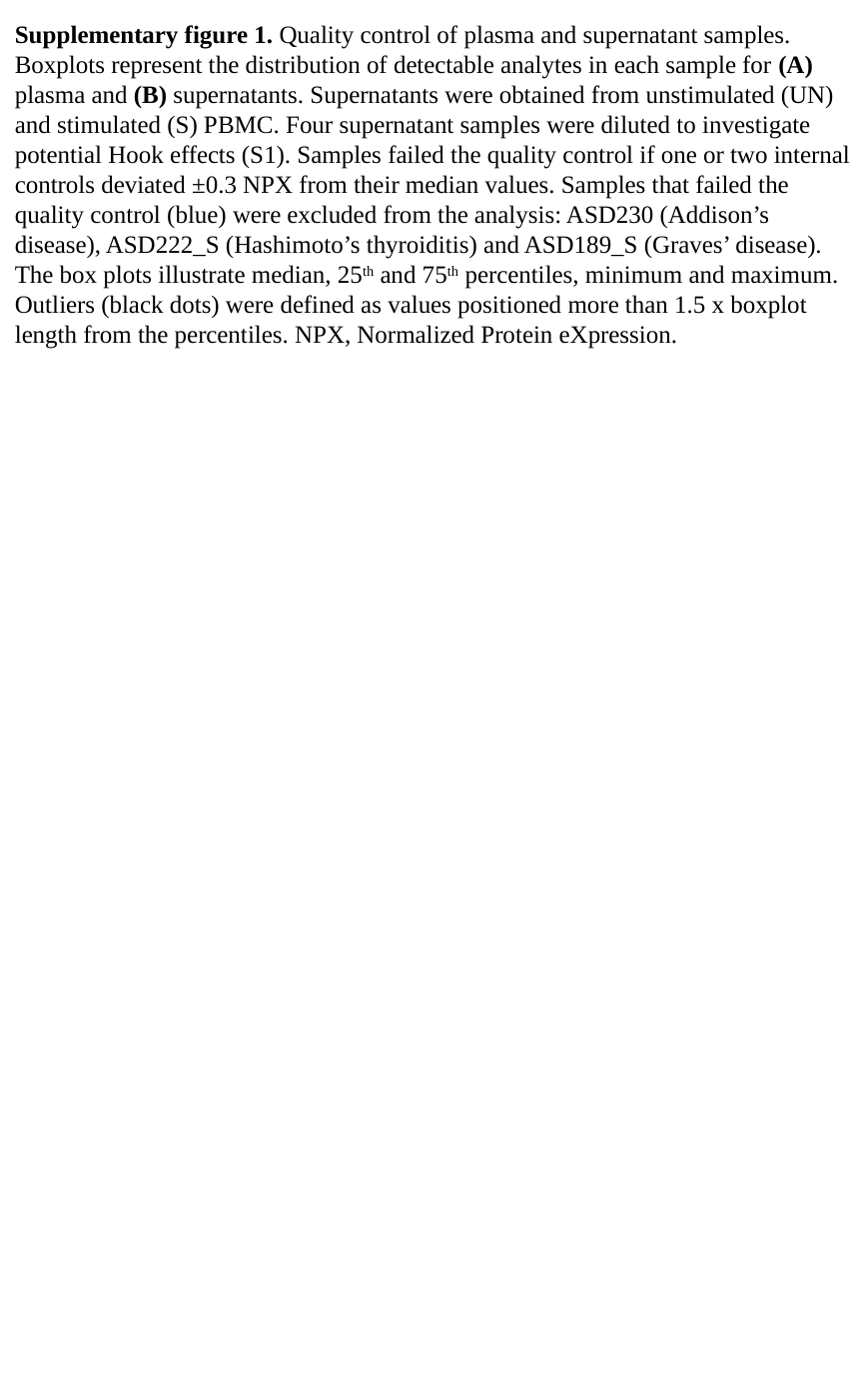

Supplementary figure 1. Quality control of plasma and supernatant samples. Boxplots represent the distribution of detectable analytes in each sample for (A) plasma and (B) supernatants. Supernatants were obtained from unstimulated (UN) and stimulated (S) PBMC. Four supernatant samples were diluted to investigate potential Hook effects (S1). Samples failed the quality control if one or two internal controls deviated ±0.3 NPX from their median values. Samples that failed the quality control (blue) were excluded from the analysis: ASD230 (Addison’s disease), ASD222_S (Hashimoto’s thyroiditis) and ASD189_S (Graves’ disease). The box plots illustrate median, 25th and 75th percentiles, minimum and maximum. Outliers (black dots) were defined as values positioned more than 1.5 x boxplot length from the percentiles. NPX, Normalized Protein eXpression.

## Slide 4
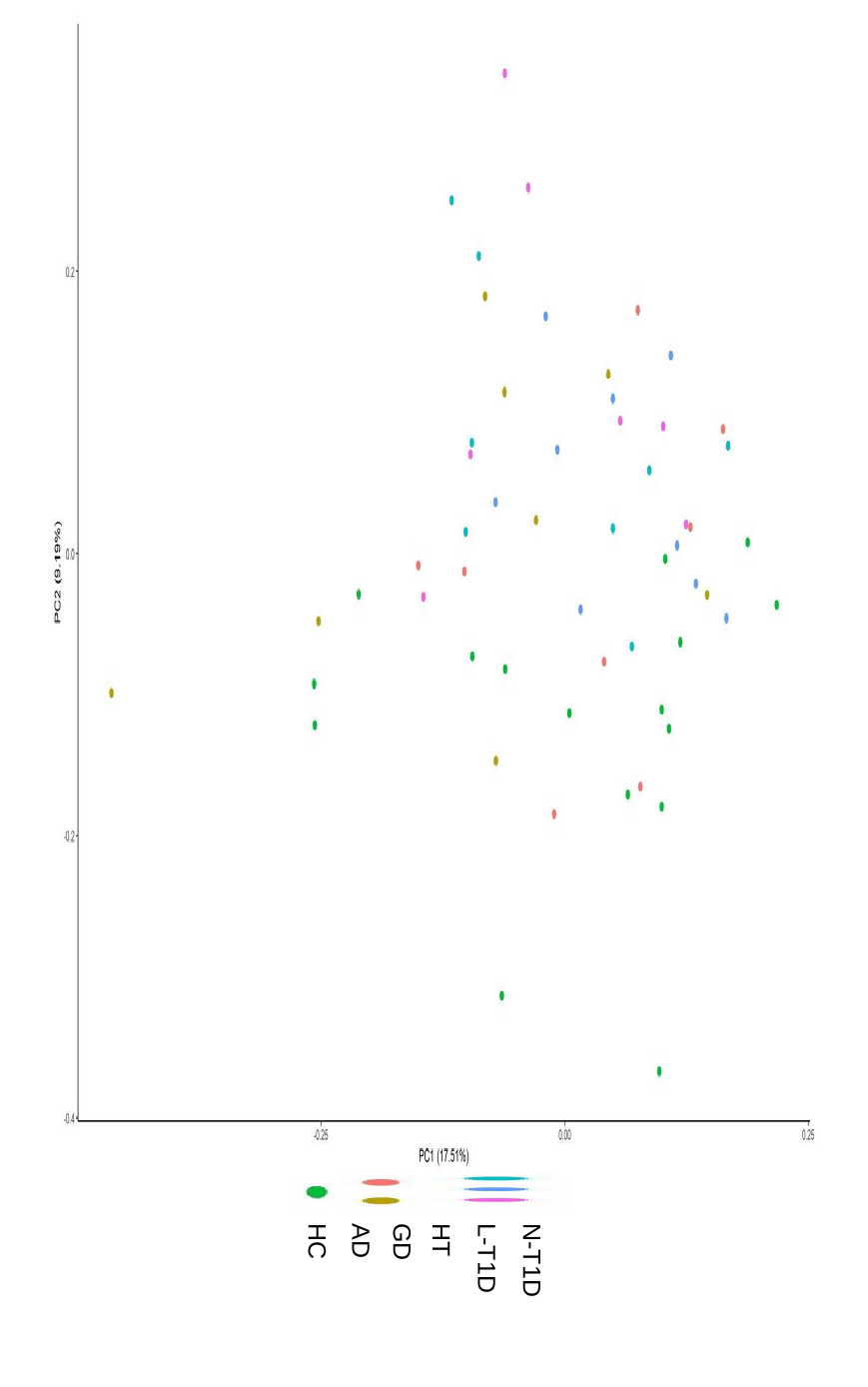

N-T1D
L-T1D
HT
GD
AD
HC

## Slide 5
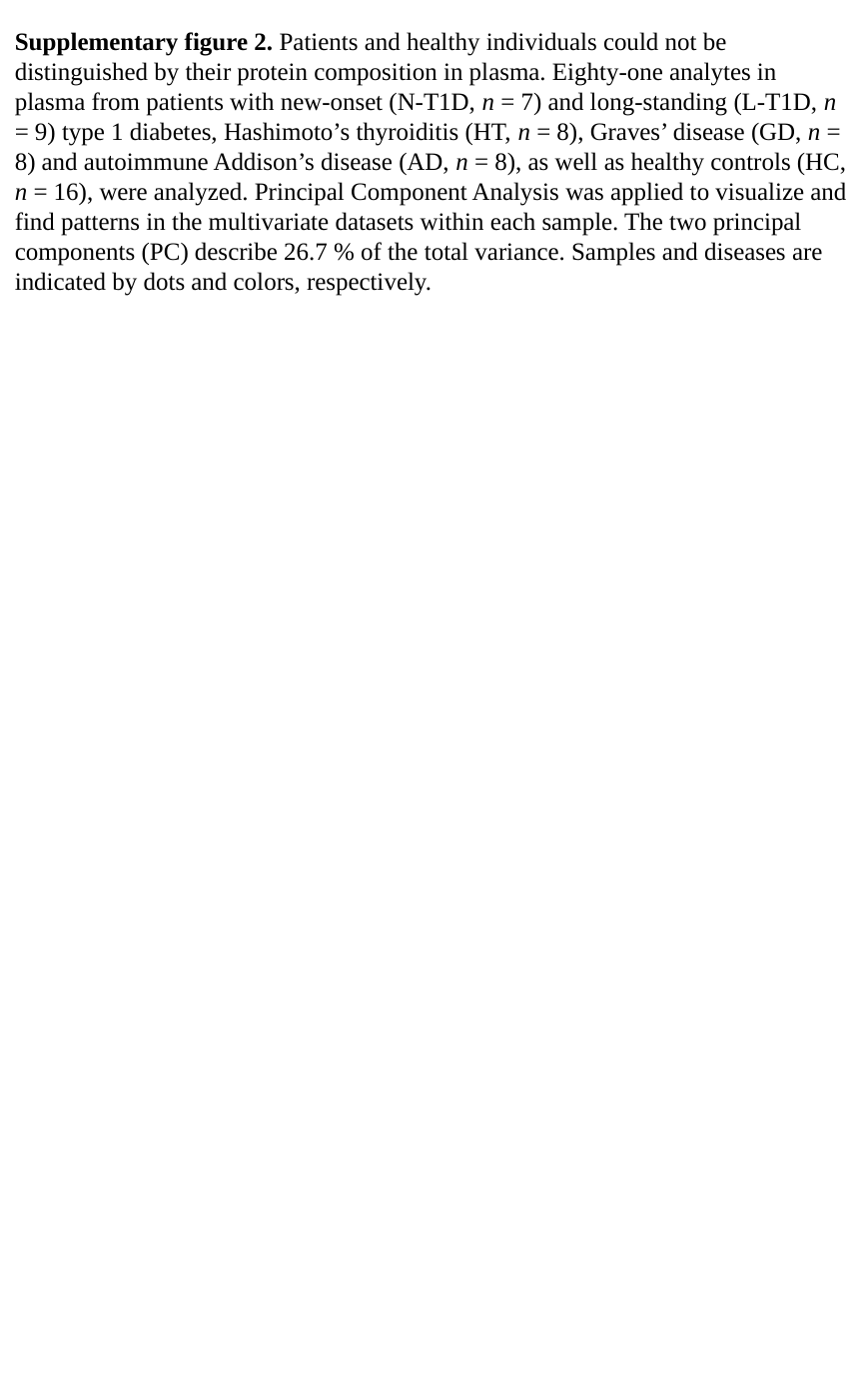

Supplementary figure 2. Patients and healthy individuals could not be distinguished by their protein composition in plasma. Eighty-one analytes in plasma from patients with new-onset (N-T1D, n = 7) and long-standing (L-T1D, n = 9) type 1 diabetes, Hashimoto’s thyroiditis (HT, n = 8), Graves’ disease (GD, n = 8) and autoimmune Addison’s disease (AD, n = 8), as well as healthy controls (HC, n = 16), were analyzed. Principal Component Analysis was applied to visualize and find patterns in the multivariate datasets within each sample. The two principal components (PC) describe 26.7 % of the total variance. Samples and diseases are indicated by dots and colors, respectively.

## Slide 6
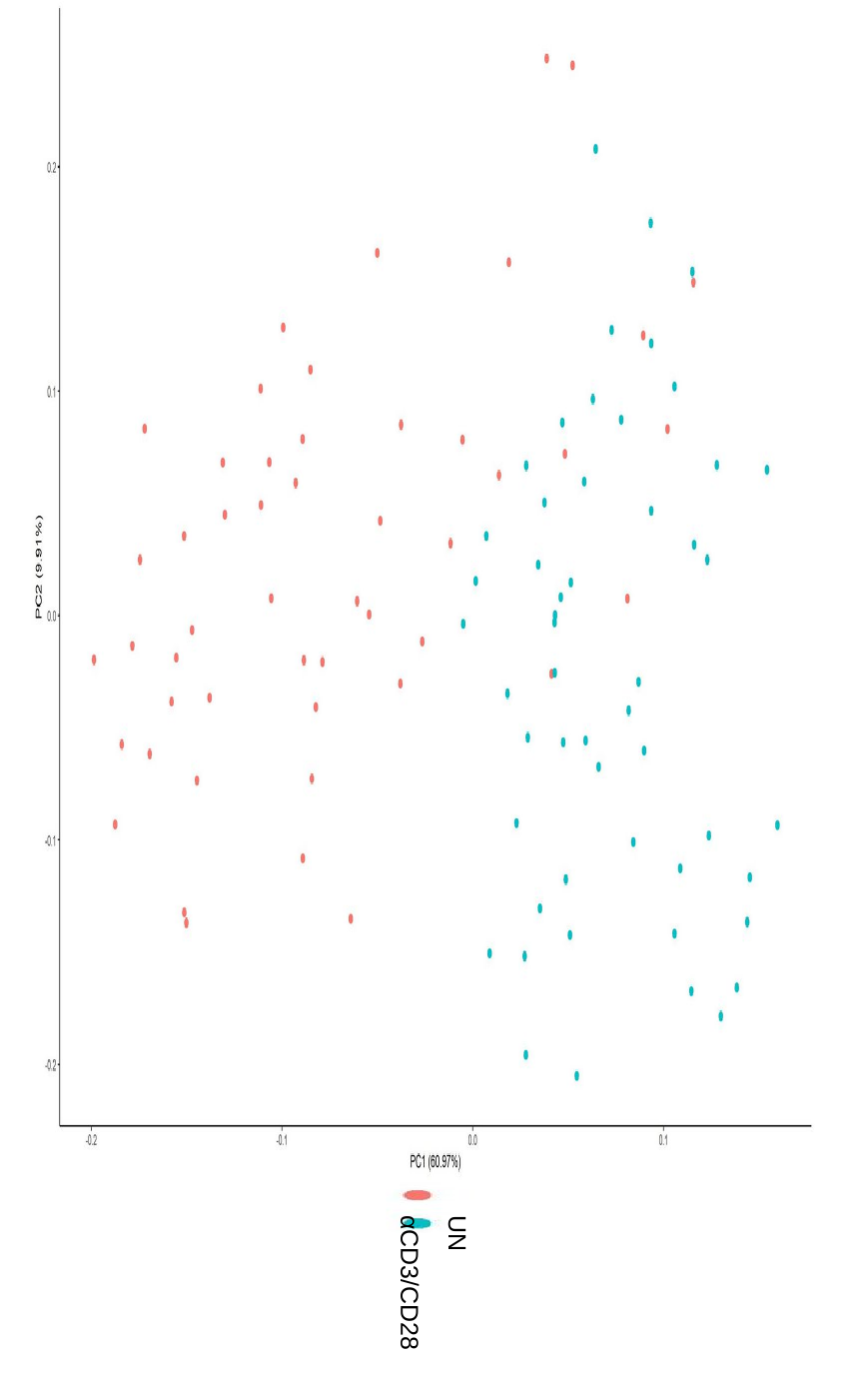

UN
αCD3/CD28

## Slide 7
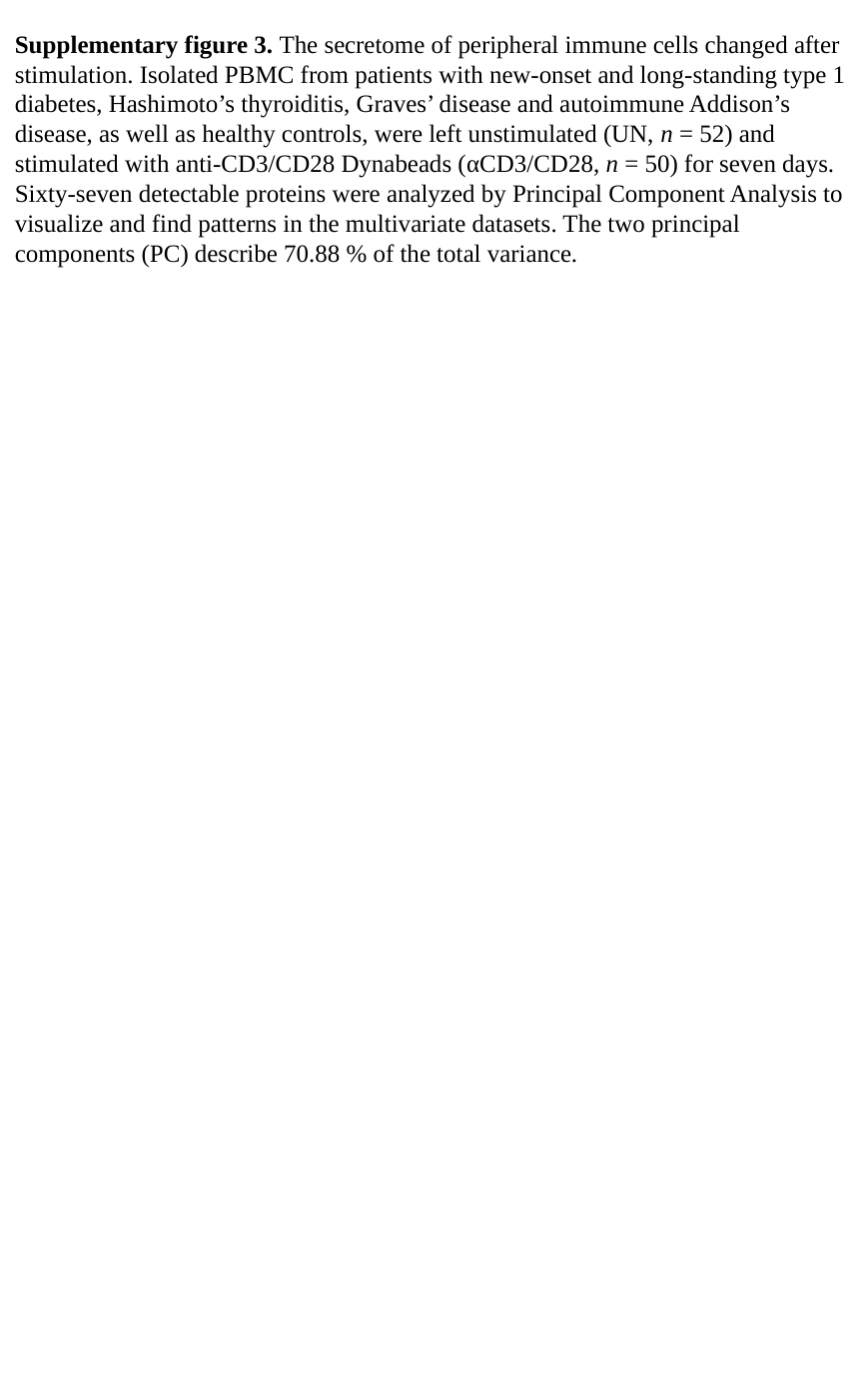

Supplementary figure 3. The secretome of peripheral immune cells changed after stimulation. Isolated PBMC from patients with new-onset and long-standing type 1 diabetes, Hashimoto’s thyroiditis, Graves’ disease and autoimmune Addison’s disease, as well as healthy controls, were left unstimulated (UN, n = 52) and stimulated with anti-CD3/CD28 Dynabeads (αCD3/CD28, n = 50) for seven days. Sixty-seven detectable proteins were analyzed by Principal Component Analysis to visualize and find patterns in the multivariate datasets. The two principal components (PC) describe 70.88 % of the total variance.

## Slide 8
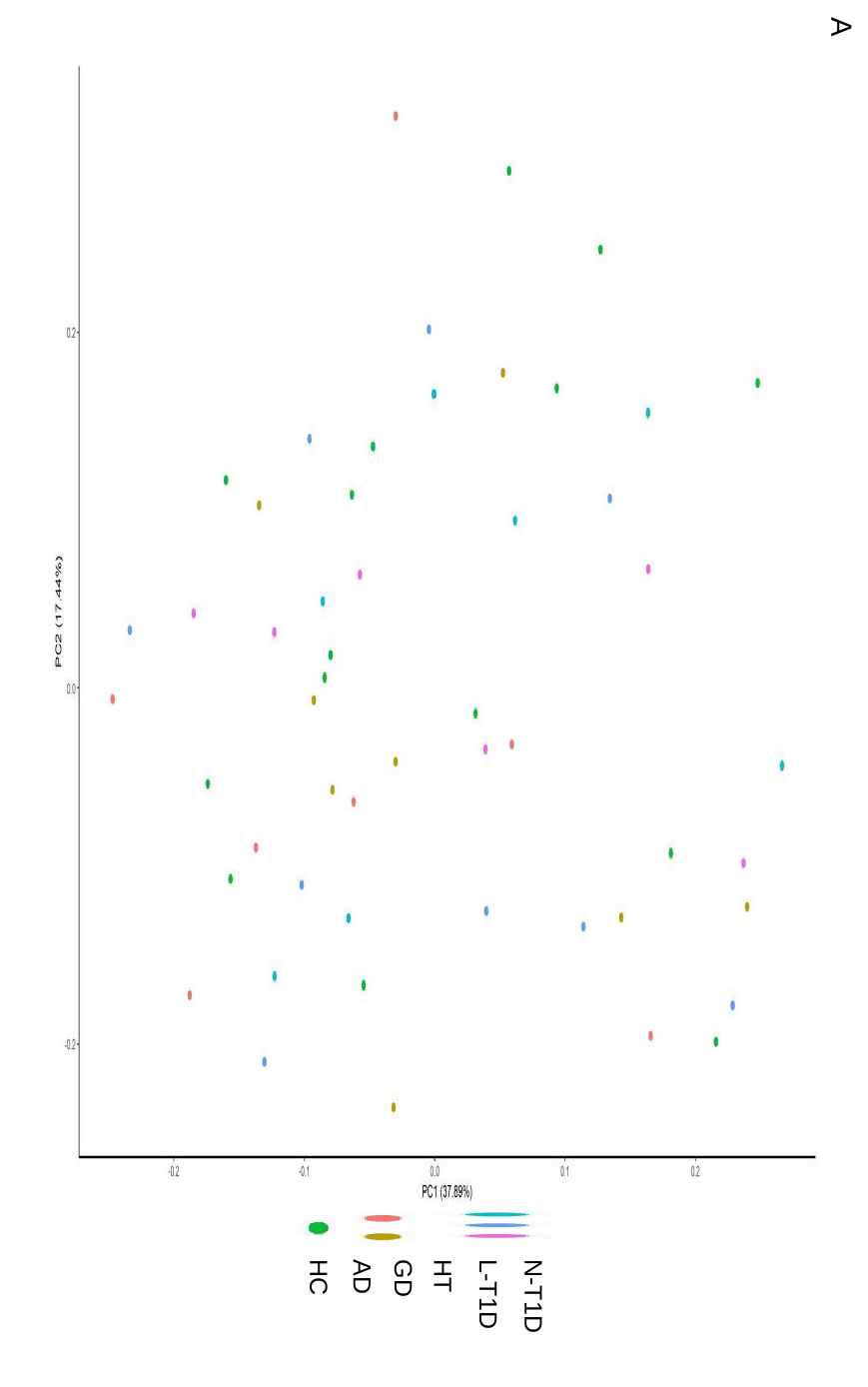

A
N-T1D
L-T1D
HT
GD
AD
HC

## Slide 9
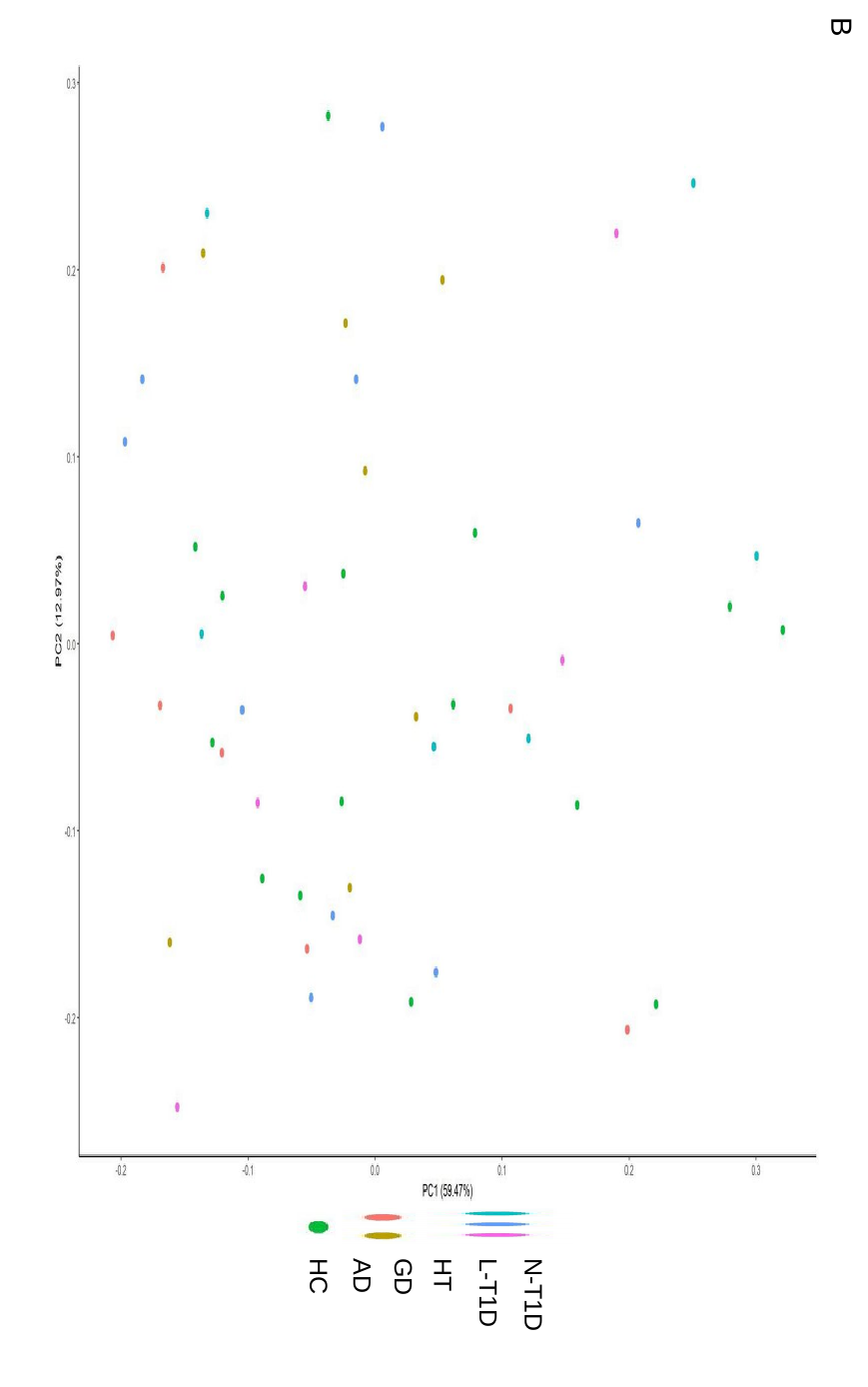

B
N-T1D
L-T1D
HT
GD
AD
HC

## Slide 10
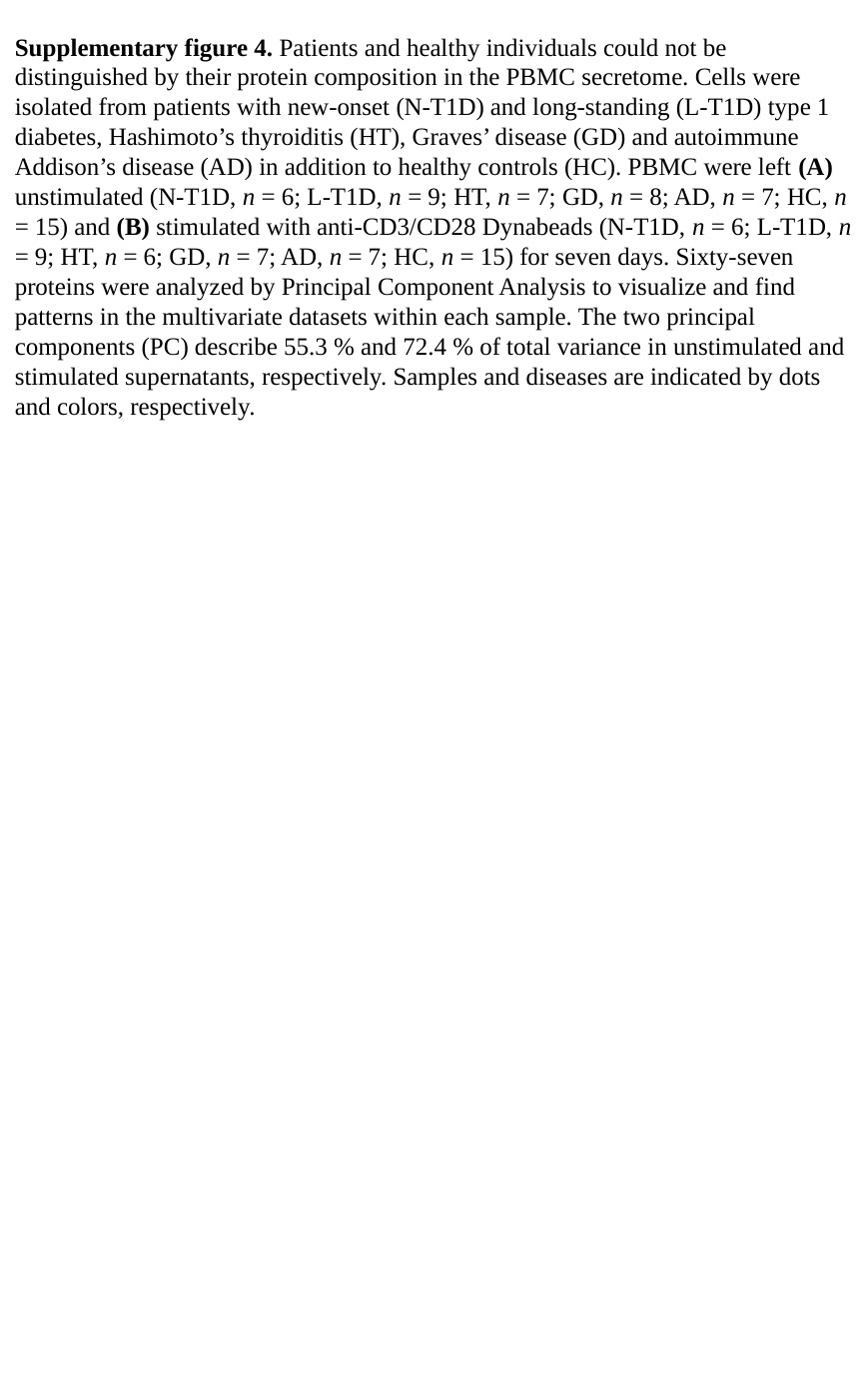

Supplementary figure 4. Patients and healthy individuals could not be distinguished by their protein composition in the PBMC secretome. Cells were isolated from patients with new-onset (N-T1D) and long-standing (L-T1D) type 1 diabetes, Hashimoto’s thyroiditis (HT), Graves’ disease (GD) and autoimmune Addison’s disease (AD) in addition to healthy controls (HC). PBMC were left (A) unstimulated (N-T1D, n = 6; L-T1D, n = 9; HT, n = 7; GD, n = 8; AD, n = 7; HC, n = 15) and (B) stimulated with anti-CD3/CD28 Dynabeads (N-T1D, n = 6; L-T1D, n = 9; HT, n = 6; GD, n = 7; AD, n = 7; HC, n = 15) for seven days. Sixty-seven proteins were analyzed by Principal Component Analysis to visualize and find patterns in the multivariate datasets within each sample. The two principal components (PC) describe 55.3 % and 72.4 % of total variance in unstimulated and stimulated supernatants, respectively. Samples and diseases are indicated by dots and colors, respectively.
