# Supplementary material for: Increased Plasma Levels of the Co-stimulatory Proteins CDCP1 and SLAMF1 in Patients With Autoimmune Endocrine Diseases
Source: Front Immunol. 2020 Aug 24;11:1916. doi: 10.3389/fimmu.2020.01916 (PMC7476208; doi:10.3389/fimmu.2020.01916)
Supplement: Supplementary file 2 [file Table_1.DOCX]

**Supplementary table 1.** Intra- and inter-assay coefficient of variance (% CV) for Olink® INFLAMMATION panel. Matrix-specific external sample controls were added in duplicates to all plates. Linear NPX-values (2^NPX^) for the external sample controls were used to calculate % CV. Average % CV and the number of analytes that fell into defined % CV intervals are stated. Reference values (Ref) for average intra- and inter-assay % CV are indicated. NA, not applicable; NPX, Normalized Protein eXpression.

|  | **Distribution** | | | | **Average % CV** |
| --- | --- | --- | --- | --- | --- |
| **Intra % CV** | **<5 %** | **5-10 %** | **10-15 %** | **>15 %** | **Ref <15 %** |
| Plasma | 48 | 37 | 6 | 1 | 6 |
| Supernatants | 66 | 22 | 3 | 1 | 5 |
| **Inter % CV** | **<10 %** | **10-20 %** | **20-30 %** | **>30 %** | **Ref <25 %** |
| Plasma |  |  |  |  | NA |
| Supernatants | 56 | 19 | 7 | 10 | 15 |

**Supplementary table 2.** Analytes included in the 92-plex Olink® INFLAMMATION panel. UniProt number as well as detectability in the matrices are indicated.

| **Analyte (Abbreviation)** | **UniProt** | **Detected in plasma?** | **Detected in supernatant?** |
| --- | --- | --- | --- |
| Adenosine Deaminase (ADA) | P00813 | Yes | Yes |
| Artemin (ARTN) | Q5T4W7 | Yes |  |
| Axin-1 (AXIN1) | O15169 | Yes |  |
| Beta-nerve growth factor (Beta-NGF) | P01138 | Yes |  |
| Caspase-8 (CASP-8) | Q14790 | Yes | Yes |
| C-C motif chemokine 3 (CCL3) | P10147 | Yes | Yes |
| C-C motif chemokine 4 (CCL4) | P13236 | Yes | Yes |
| C-C motif chemokine 19 (CCL19) | Q99731 | Yes | Yes |
| C-C motif chemokine 20 (CCL20) | P78556 | Yes | Yes |
| C-C motif chemokine 23 (CCL23) | P55773 | Yes | Yes |
| C-C motif chemokine 25 (CCL25) | O15444 | Yes |  |
| C-C motif chemokine 28 (CCL28) | Q9NRJ3 | Yes | Yes |
| CD40L receptor (CD40) | P25942 | Yes | Yes |
| CUB domain-containing protein 1 (CDCP1) | Q9H5V8 | Yes | Yes |
| C-X-C motif chemokine 1 (CXCL1) | P09341 | Yes | Yes |
| C-X-C motif chemokine 5 (CXCL5) | P42830 | Yes | Yes |
| C-X-C motif chemokine 6 (CXCL6) | P80162 | Yes | Yes |
| C-X-C motif chemokine 9 (CXCL9) | Q07325 | Yes | Yes |
| C-X-C motif chemokine 10 (CXCL10) | P02778 | Yes | Yes |
| C-X-C motif chemokine 11 (CXCL11) | O14625 | Yes | Yes |
| Cystatin D (CST5) | P28325 | Yes | Yes |
| Delta and Notch-like epidermal growth factor-related receptor (DNER) | Q8NFT8 | Yes | Yes |
| Eotaxin (CCL11) | P51671 | Yes |  |
| Eukaryotic translation initiation factor 4E-binding protein 1 (4E-BP1) | Q13541 | Yes | Yes |
| Fibroblast growth factor 21 (FGF-21) | Q9NSA1 | Yes |  |
| Fibroblast growth factor 23 (FGF-23) | Q9GZV9 | Yes |  |
| Fibroblast growth factor 5 (FGF-5) | Q8NF90 |  |  |
| Fibroblast growth factor 19 (FGF-19) | O95750 | Yes |  |
| Fms-related tyrosine kinase 3 ligand (Flt3L) | P49771 | Yes | Yes |
| Fractalkine (CX3CL1) | P78423 | Yes |  |
| Glial cell line-derived neurotrophic factor (GDNF) | P39905 | Yes |  |
| Hepatocyte growth factor (HGF) | P14210 | Yes | Yes |
| Interferon gamma (IFN-gamma) | P01579 |  | Yes |
| Interleukin-1 alpha (IL-1 alpha) | P01583 |  | Yes |
| Interleukin-2 (IL-2) | P60568 |  |  |
| Interleukin-2 receptor subunit beta (IL-2RB) | P14784 |  | Yes |
| Interleukin-4 (IL-4) | P05112 | Yes | Yes |
| Interleukin-5 (IL5) | P05113 | Yes | Yes |
| Interleukin-6 (IL6) | P05231 | Yes | Yes |
| Interleukin-7 (IL-7) | P13232 | Yes |  |
| Interleukin-8 (IL-8) | P10145 | Yes | Yes |
| Interleukin-10 (IL10) | P22301 | Yes | Yes |
| Interleukin-10 receptor subunit alpha (IL-10RA) | Q13651 | Yes | Yes |
| Interleukin-10 receptor subunit beta (IL-10RB) | Q08334 | Yes | Yes |
| Interleukin-12 subunit beta (IL-12B) | P29460 | Yes | Yes |
| Interleukin-13 (IL-13) | P35225 | Yes | Yes |
| Interleukin-15 receptor subunit alpha (IL-15RA) | Q13261 | Yes |  |
| Interleukin-17A (IL-17A) | Q16552 | Yes | Yes |
| Interleukin-17C (IL-17C) | Q9P0M4 | Yes |  |
| Interleukin-18 (IL-18) | Q14116 | Yes |  |
| Interleukin-18 receptor 1 (IL-18R1) | Q13478 | Yes | Yes |
| Interleukin-20 (IL-20) | Q9NYY1 |  |  |
| Interleukin-20 receptor subunit alpha (IL-20RA) | Q9UHF4 | Yes |  |
| Interleukin-22 receptor subunit alpha-1 (IL-22 RA1) | Q8N6P7 | Yes |  |
| Interleukin-24 (IL-24) | Q13007 | Yes |  |
| Interleukin-33 (IL-33) | O95760 |  |  |
| Latency-associated peptide transforming growth factor beta-1 (LAP TGF-beta-1) | P01137 | Yes | Yes |
| Leukemia inhibitory factor (LIF) | P15018 |  | Yes |
| Leukemia inhibitory factor receptor (LIF-R) | P42702 | Yes |  |
| Macrophage colony-stimulating factor 1 (CSF-1) | P09603 | Yes | Yes |
| Matrix metalloproteinase-1 (MMP-1) | P03956 | Yes | Yes |
| Matrix metalloproteinase-10 (MMP-10) | P09238 | Yes | Yes |
| Monocyte chemotactic protein 1 (MCP-1) | P13500 | Yes | Yes |
| Monocyte chemotactic protein 2 (MCP-2) | P80075 | Yes | Yes |
| Monocyte chemotactic protein 3 (MCP-3) | P80098 | Yes | Yes |
| Monocyte chemotactic protein 4 (MCP-4) | Q99616 | Yes | Yes |
| Natural killer cell receptor 2B4 (CD244) | Q9BZW8 | Yes | Yes |
| Neurotrophin-3 (NT-3) | P20783 | Yes |  |
| Neurturin (NRTN) | Q99748 |  |  |
| Oncostatin-M (OSM) | P13725 | Yes | Yes |
| Osteoprotegerin (OPG) | O00300 | Yes | Yes |
| Programmed cell death 1 ligand 1 (PD-L1) | Q9NZQ7 | Yes | Yes |
| Protein S100-A12 (EN-RAGE) | P80511 | Yes | Yes |
| Signaling lymphocytic activation molecule (SLAMF1) | Q13291 | Yes | Yes |
| SIR2-like protein 2 (SIRT2) | Q8IXJ6 | Yes | Yes |
| STAM-binding protein (STAMBP) | O95630 | Yes | Yes |
| Stem cell factor (SCF) | P21583 | Yes | Yes |
| Sulfotransferase 1A1 (ST1A1) | P50225 | Yes | Yes |
| T cell surface glycoprotein CD6 isoform (CD6) | Q8WWJ7 | Yes | Yes |
| T-cell surface glycoprotein CD5 (CD5) | P06127 | Yes | Yes |
| T-cell surface glycoprotein CD8 alpha chain (CD8A) | P01732 | Yes | Yes |
| Thymic stromal lymphopoietin (TSLP) | Q969D9 |  |  |
| TNF-beta (TNFB) | P01374 | Yes | Yes |
| TNF-related activation-induced cytokine (TRANCE) | O14788 | Yes | Yes |
| TNF-related apoptosis-inducing ligand (TRAIL) | P50591 | Yes | Yes |
| Transforming growth factor alpha (TGF-alpha) | P01135 | Yes | Yes |
| Tumor necrosis factor (Ligand) superfamily, member 12 (TWEAK) | O43508 | Yes | Yes |
| Tumor necrosis factor (TNF) | P01375 |  | Yes |
| Tumor necrosis factor ligand superfamily member 14 (TNFSF14) | O43557 | Yes | Yes |
| Tumor necrosis factor receptor superfamily member 9 (TNFRSF9) | Q07011 | Yes | Yes |
| Urokinase-type plasminogen activator (uPA) | P00749 | Yes | Yes |
| Vascular endothelial growth factor A (VEGF-A) | P15692 | Yes | Yes |
